# Pelizaeus-Merzbacher Disease as a Cause of Early-Onset Developmental Delay: A Case Report

**DOI:** 10.7759/cureus.82308

**Published:** 2025-04-15

**Authors:** Michela Galea, Miriana Cocker, Doriette Soler, Esther Bezzina

**Affiliations:** 1 Paediatrics, Mater Dei Hospital, Msida, MLT; 2 Radiology, Mater Dei Hospital, Msida, MLT

**Keywords:** global developmental delay (gdd), hypomyelination, leukodystrophy, pelizaeus-merzbacher disease, plp1 gene

## Abstract

Pelizaeus-Merzbacher disease (PMD) is a rare X-linked hypomyelinating leukodystrophy disorder caused by a mutation in the proteolipid protein 1 (PLP1) gene which is responsible for myelin formation in the central nervous system. We report a case of PMD in a male patient who initially presented with developmental delay at three months and was ultimately diagnosed at 10 years and 7 months of age. We aim to describe the initial presentation, clinical course of PMD and the investigations that aid in diagnosis so that future cases may be identified earlier. During the investigative workup of our patient, a deletion of exon 16 - an extremely rare heterozygous nucleotide variation, c.330C>T (p.D110D) - was identified, the pathogenicity of which has not been previously documented in the literature. In our report, we also aim to highlight the potential use of biotin in improving symptoms in such patients as there is currently no curative treatment available.

## Introduction

Pelizaeus-Merzbacher disease (PMD) is a rare leukodystrophy disorder, with a worldwide incidence of 1 in 90,000 to 1 in 750,000 live births [1]. It was first described by Pelizaeus in 1885 then further documented in 1990 by Merzbacher when he discovered a familial cluster of male members sharing the same constellation of clinical features: developmental delay, spastic paraparesis, ataxia and nystagmus [2,3]. It is caused by a mutation in the proteolipid protein 1 (PLP1) gene located on chromosome Xq22, which is responsible for oligodendrocyte development and myelin formation within the central nervous system [1]. There are various possible mutations to the PLP1 gene that might occur, with PLP1 gene duplication being the most common, occurring in 50%-75% of cases. Being an X-linked recessive condition, it commonly affects males while females are usually carriers [4].

PMD usually presents with neurological impairment in infancy or early childhood, with varying degrees of clinical severity secondary to its genetically heterogeneous nature. In fact, it is subdivided into three major subtypes: connatal, classic and transitional PMD, depending on the age of presentation and the patient’s motor skills. Connatal PMD, caused by PLP1 missense mutations, is the most severe subtype, with patients presenting with neonatal hypotonia, nystagmus and respiratory distress very early on, within a few weeks of birth. Such patients do not usually survive past adolescence. Classic PMD is the least aggressive subtype, presenting with a less severe developmental delay before one year of age, with better cognition, speech and motor skills compared to connatal PMD. On the other hand, transitional PMD is the intermediate form, having features of both classic and connatal subtypes. Nonetheless, all three subtypes are characterised by significant morbidity due to progressive neurodegeneration caused by the complete absence of myelin, which ultimately leads to early mortality [1,5].

Hypomyelination with diffuse cerebral and cerebellar atrophy on magnetic resonance imaging (MRI), as well as the identification of the PLP1 gene mutation on whole exome sequencing, are pivotal in the early diagnosis of PMD to differentiate it from other neurodegenerative disorders [1]. Prognosis is poor because, despite medical advancements, there is still no cure for PMD, and symptomatic control in combination with a multidisciplinary team approach remains the mainstay of treatment in providing holistic care for such patients.

## Case presentation

We report a case of an 11-year-old boy born to non-consanguineous parents at term via caesarean section, weighing 2.9 kg. There were no significant antenatal or postnatal issues. There was no family history of neurological disorders. 

**Figure 1:**
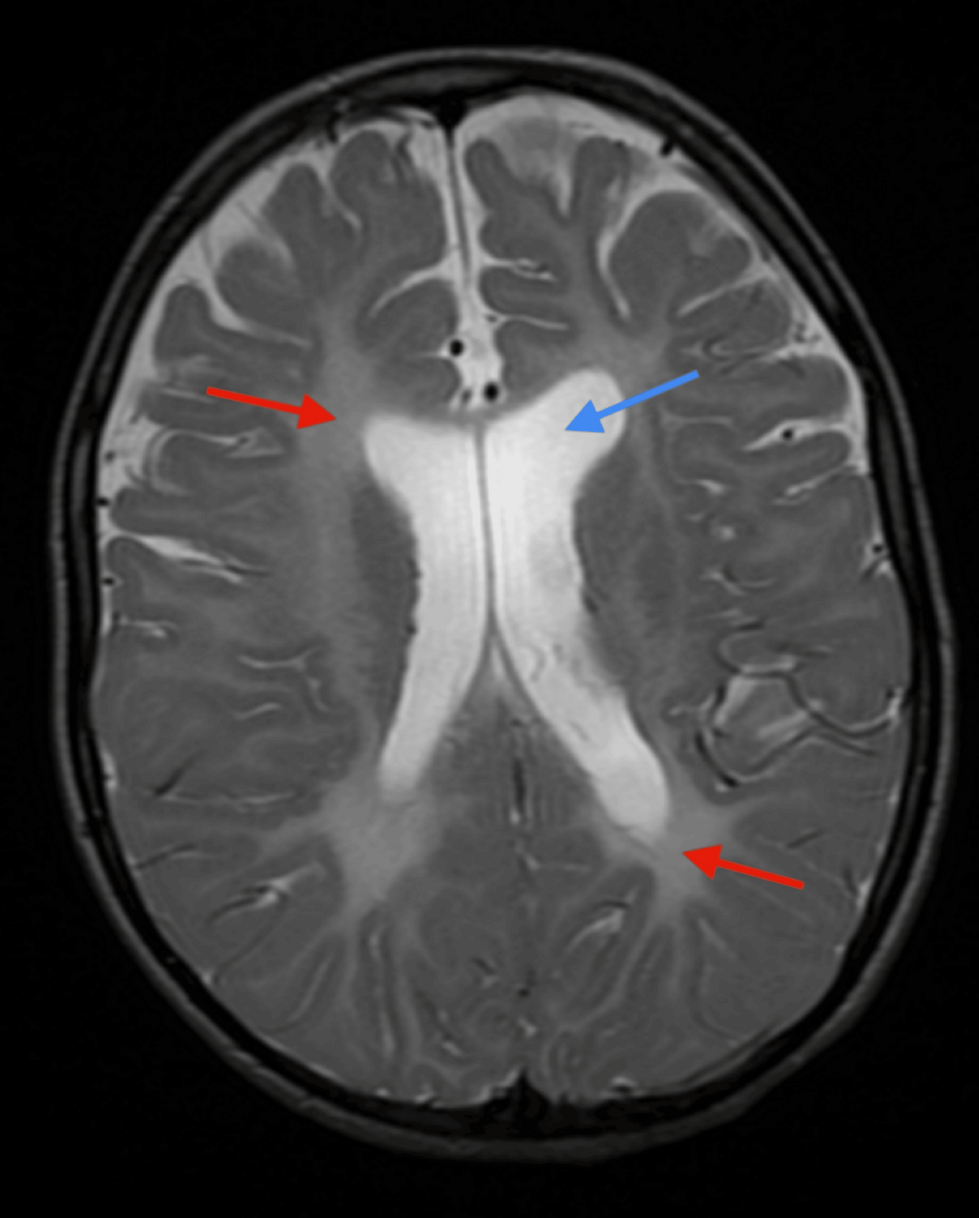
Axial T2-weighted imaging at the level of the basal ganglia The basal ganglia are normal in this case. Prominent perivascular spaces were noted. There is diffuse bilateral white matter T2 hyperintensity (red arrows) and relative cerebral volume loss of the frontal lobes with dilatation of the lateral and third ventricles (blue arrow).

In the early weeks of life, the child was reaching age-appropriate milestones. At six weeks of age, he had a social smile, startled by loud noise and had normal newborn reflexes. However, at three months of age, he presented with signs of global developmental delay including a lack of head control, hypotonia, irritability and restlessness. Nystagmus was present with an inability to fix and follow. No cataracts were evident on the ophthalmic exam and there were no dysmorphic features or stigmata. Considering the early presentation of developmental delay and irritability, the initial differential diagnosis was Krabbe disease. Hence, sequencing of exons 1 to 17 was performed to exclude a GALC gene mutation. Results showed a deletion of exon 16 - an extremely rare heterozygous nucleotide variation, c.330C>T (p.D110D), whose grade of pathogenicity has not been previously identified in the literature. Cerebral MRI showed no identifiable myelination signs in the infra- and supratentorial regions with cerebellar and putamen signal anomalies corresponding to neurodegenerative disease (Figure 1).

**Figure 2:**
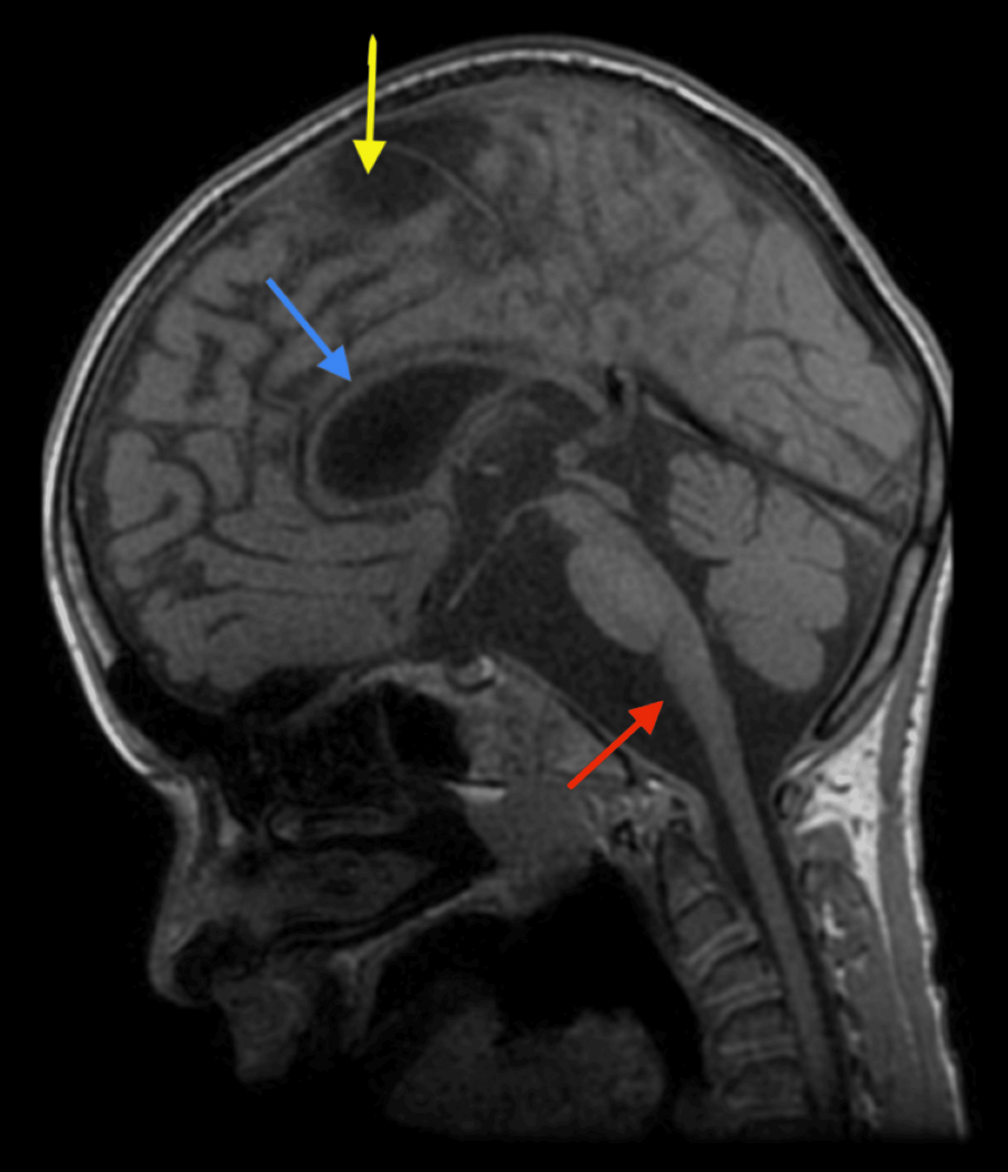
Sagittal T1-weighted imaging The corpus callosum is severely thinned throughout (blue arrow). The brainstem is slender, with large cerebrospinal fluid (CSF) spaces, particularly anterior to the medulla oblongata (red arrow). The foramen magnum is normally sized. An incidental note is made of a right parietal arachnoid cyst at the vertex (yellow arrow).

Hence, a full metabolic screen was performed. All blood investigations including a complete blood count, urea, creatinine, electrolytes, liver and thyroid function tests, albumin, ammonia and haematinics (including a vitamin B level) were within normal limits. Cerebrospinal fluid amino acid levels were normal with a low lactate level. Electroencephalography (EEG) showed signs of mild non-specific encephalopathy with background activity containing symmetrical semi-synchronous medium amplitude (20-40 µV) theta activity mixed with fast activity over both hemispheres and few delta transients. Repeat MRI at two years and three months of age showed a relatively stable aspect of the white matter substance with diffuse signal modification involving the corpus callosum and basal nuclei (Figure 2). This correlated clinically as no new developmental skills were observed.

A trial of B1, B8 and high-dose B7 vitamin supplementation was attempted at four years and four months of age. There was a notable gain of some head control and the ability to take steps with support. Improvement was also noted on his EEG. This showed background tracing with reactive theta/delta rhythm while closing and opening the eyes with 70 µV/mm maximum amplitude, 6 c/sec frequency located in the posterior derivations (parieto-occipital and temporo-occipital) and low amplitude, high-frequency beta rhythm in the frontal derivations with no spontaneous epileptiform discharges. Electroretinogram (ERG) showed improved nerve conduction in the previously identified bilateral optic nerve atrophy, six months after starting B vitamins. No regression was observed in vitamin B1 withdrawal and reduction in vitamin B8 dose.

By five years of age, he had persistent moderate general hypotonia which was more marked axially, with clonus and brisk reflexes being more pronounced in the lower limbs. He was able to turn from supine to prone when laid flat and lift his head when placed prone. He had difficulty gesturing and grabbing objects, was unable to sit unattended and no change in walking ability was noted. He had no hearing difficulties and was able to understand words addressed to him. However, speech delay was pronounced, being able to utter only a few words and mainly communicated by facial expressions to show emotions and crying to express needs. Rotary and horizontal nystagmus as well as inconsistent visual fixation persisted but the patient was able to look at parents when spoken to, had better eye following and attempts at maintaining eye contact were observed. Repeat imaging showed bilateral optic nerve atrophy with a cessation of the cerebral degradation process but remyelination was not observed when compared to previous imaging. 

Whole exome sequencing at 10 years and 7 months of age confirmed the presence of a homozygous pathogenic variant in the PLP1 gene, c.696+1G>A, diagnostic of X-linked PMD-hypomyelination leukodystrophy 1 (HLD1).

At 11 years, the patient had severe learning difficulties, optic atrophy, dystonic movements controlled on clonidine and stable thoracolumbar scoliosis. He had an alert, hypotonic face and lean build with poor muscle mass. He had truncal hypotonia with few spontaneous movements and spasticity, more pronounced in the lower limbs. Although developmental delay was noted at three months of age, he acquired skills since then and made some developmental progress. He was more alert, communicated mainly with different facial expressions, uttered few words, showed empathy and gave different cues for needs. He did not voluntarily reach out to grab objects but was able to hold objects when placed in his hands. He mobilised mainly using a wheelchair but could stand and walk a few steps using a frame. With regards to systemic examination of his cardiovascular, respiratory and abdominal systems, this remained unremarkable and relatively stable throughout the years. He did not have any history of aspiration pneumonia and did not require any respiratory support. However, weight gain remained an issue. He continued to be spoon-fed pureed food due to swallowing issues and had episodes of back arching and hyperextension related to feeds which improved with esomeprazole. He was also chronically constipated, requiring laxatives long-term. His gastrointestinal issues were attributed to gastrointestinal dysmotility secondary to his neurodevelopmental delay. He also had primary incontinence with no reported history of recurrent urinary tract infections. He continued to be followed up regularly by a multidisciplinary team. 

## Discussion

PMD depicts a wide spectrum of varying clinical severity owing to its genetic heterogeneity [4]. Patients with PMD, however, share some general characteristics irrespective of clinical subtype, as shown by the results from a case series study performed on seven Colombian male patients aged between 6 months and 16 years. All seven patients had developmental delay, whereas 85.7% had spasticity, 71.4% had cerebellar findings on examination, 57% were hypotonic and nystagmus was reported in 28.7% [6].

Our case report describes a patient with classic PMD, which is the most common subtype. It typically presents within the first year of life with developmental delay, with milestones being either slowly acquired or unachieved. The child in our case presented at three months of age. Such patients eventually develop spasticity and ataxia, with the development of choreoathetoid movements and seizures later on. Despite this, they are still able to walk, although with some degree of support, and cognition is maintained with the ability to communicate. They also have a history of nystagmus, which usually resolves, and optic atrophy [1]. PMD mainly affects the central nervous system, with limited involvement of the peripheral nervous system. These features are consistent with signs and symptoms in our patient. Although the prognosis is poor, patients with classic PMD usually live up to young adulthood. This is in contrast to the more aggressive connatal PMD subtype, whereby patients usually present with significant cognitive impairment and developmental delay, with inability to ambulate, severe hypotonia, stridor and feeding issues. They usually do not survive past early childhood [7].

Our case report showed that apart from whole-exome gene sequencing, imaging also plays a pivotal role in diagnosing PMD and distinguishing it from other diseases. MRI in PMD patients shows hypomyelination with an increase in T2 signalling in the supratentorial region and hypointensity on T1 weighted images involving the internal capsule, optic radiation and proximal corona radiata [8]. This correlates with the MRI of our patient, which showed diffuse significant hypomyelination with involvement of the basal nuclei and a thin corpus callosum. A study conducted by Sumida et al. on 19 genetically proven PMD male patients demonstrated that although the spectrum of imaging findings on MRI is wide, clinical severity may be presumed when there is signal intensity of the brainstem and corticospinal tract of the internal capsule [9]. The degree of gross motor delay is linked to the extent of corpus callosum and cerebellar atrophy as observed in the study performed by Sarret et al. on 35 patients with PLP-1 mutations. Such findings were clearly evident on our patient’s MRI. White matter deficit remains stable, until at least adolescent age [5]. 

The presentation of PMD may be similar to other leukodystrophies such as Krabbe disease. This is an autosomal recessive lysosomal storage disorder characterised by a deficiency in galactocerebrosidase resulting in demyelination [10]. Krabbe disease was, however, excluded following exon sequencing of exons 1 to 17 when the presence of the GALC gene mutation was not detected. PMD can also mimic other neurometabolic disorders such as Leigh syndrome. The latter was initially considered in our patient following putamen signal anomalies on MRI. Leigh syndrome is an autosomal recessive condition which typically causes progressive neurodegeneration. On MRI it is characterised by symmetrical lesions in the brain stem and basal ganglia, predominantly the substantia nigra and putamen [11]. However, this diagnosis became less likely in our patient due to a lack of progressive loss in cognition and of previously acquired motor skills. Biotin-thiamine responsive basal ganglia disease and biotinidase deficiency were also two other differential diagnoses that were considered as there was a noticeable improvement in symptoms in our patient when trialled on biotin and thiamine. However, these were later refuted when thiamine was omitted and there was no observed regression as would have otherwise occurred in these two conditions. 

There is currently no curative treatment for PMD. Early supportive and symptomatic management of such patients via a multidisciplinary team approach can improve both morbidity and reduce complications [12]. These patients may require anti-epileptic medications for seizure control and muscle relaxants like baclofen to treat spasticity. Research studies on antisense gene therapy and stem cell transplantation show promising results but these have only been tested on genetically modified mice [4,13]. Our patient continued biotin treatment long term with symptom amelioration including attainment of some developmental skills as well as objective improvement on EEG, ERG and nerve conduction studies. Although the mechanism of action of biotin in PMD has not yet been studied in the literature, it may be hypothesised that this improvement is due to the action of biotin as an essential co-factor in long-chain fatty acid synthesis and generation of adenosine triphosphate (ATP), in turn affecting the process of myelination [14]. This may potentially represent an opportunity for further research. 

## Conclusions

Our case illustrates that although PMD is a rare condition, it should be considered as a differential diagnosis when a patient presents with global developmental delay at an early age, especially if male, considering PMD is an X-linked condition. It should be clinically suspected when there is evidence of hypotonia, nystagmus, spasticity and ataxia on examination. Diffuse hypomyelination demonstrated by hyperintensity on T2 weighted images, as well as cerebral and cerebellar atrophy on MR brain, are other indicators of PMD which also aid in determining clinical severity. Whole genome sequencing is also important to identify the PLP1 gene mutation in order to help establish the PMD subtype. Our case has shown that PMD can be easily misdiagnosed if there is no clinical suspicion, especially when trials of treatment options result in symptomatic improvement, as occurred in our case when the child was initially trialled on thiamine. This highlights the need for increased awareness regarding this condition because prompt diagnosis through genetic testing results in early intervention that might improve the quality of life of such patients. Although current treatment is mainly supportive through symptomatic control, there is the potential for stem cell therapy and PLP1 gene suppression as future novel therapies for this debilitating condition. The use of biotin, as illustrated in our case, may also be another option in aiding symptomatic improvement although further research is required.
